# Tetravalent microprojection-based dengue chimeric virus vaccine raises potent neutralising antibodies in mice

**DOI:** 10.1038/s41541-025-01297-5

**Published:** 2025-11-21

**Authors:** Jovin J. Y. Choo, Christopher L. D. McMillan, Connor A. P. Scott, Jessica J. Harrison, Daniel Watterson, Roy A. Hall, Paul R. Young, Jody Hobson-Peters, David A. Muller

**Affiliations:** 1 School of Chemistry and Molecular Biosciences, The University of Queensland, Brisbane, QLD Australia; 2 Australian Infectious Diseases Research Centre, Global Virus Network Centre of Excellence, Brisbane, QLD Australia

**Keywords:** Biotechnology, Immunology, Microbiology

## Abstract

Dengue virus (DENV) is endemic throughout the tropical regions of the world. Due to the risk posed to the population living in dengue-endemic areas, the development of a dengue vaccine has been considered a high priority by the WHO for the past 50 years. The development of a new chimeric viral platform, based on the insect-specific orthoflavivirus, Binjari virus (BinJV) has facilitated the production of multiple orthoflavivirus vaccine candidates. This study describes the evaluation of four candidate chimeric dengue virus vaccines (BinJ/DENV-prME) delivered as either a monovalent or tetravalent vaccine formulations via an alternative delivery method, the high-density microarray patch (HD-MAP). These chimeric viruses elicited potent neutralising antibodies against both homologous and heterologous serotypes and were raised to equal levels, with no immunodominance observed for any serotype. When coupled with the HD-MAP, enhanced antibody kinetics were observed, resulting in higher levels of neutralising antibodies elicited following a single dose.

## Introduction

Dengue virus (DENV) is found throughout the tropical regions of the world, with 3 billion people living under the threat of DENV infection due to the broad geographic distribution^1^. The distribution of DENV infections colocalises with its mosquito vectors. *Aedes aegypti*, the primary vector, is responsible for transmission in tropical regions of Asia, Africa, Australia, America, the Middle East and the South Pacific^1–4^. While outbreaks in temperate regions of the USA and Europe are attributed to the secondary vector, the *Aedes albopictus*^3^. DENV has four antigenically distinct serotypes, and infection with DENV can cause a range of clinical outcomes, ranging from dengue fever to severe disease such as dengue with haemorrhage or shock^3^. Whilst lifelong immunity is observed for the infecting serotype post-primary infection, cross-protection to heterologous serotypes during secondary infection is limited, and can increase the risk of severe disease^5^. Due to the risk of severe disease associated with secondary DENV infection, the ideal dengue vaccine should be tetravalent and protect against all serotypes without priming for severe disease post-vaccination^6^. As there are no therapeutic interventions to treat dengue-related disease, new vaccine strategies are urgently needed to reduce the global burden of disease associated with this debilitating disease.

The chimeric virus system based on the insect-specific orthoflavivirus (ISF), Binjari virus (BinJV), has been used for the development of several orthoflaviviral vaccine candidates^7–11^. These chimeric virions are made by exchanging the prME genes of the BinJV with those of pathogenic vertebrate-infecting orthoflaviviruses. As the chimeric viruses retain the phenotype of the insect-specific BinJV, they cannot replicate in vertebrate cells, yet replicate to high titres in mosquito cell lines^9^. These chimeric viruses also structurally mimic their parental virus with authentic presentation of quaternary epitopes necessary for inducing potent neutralising antibodies^9,12^. With their inability to replicate in mammalian cells, this broadens the potential of using the chimeric viruses as a vaccine candidate, as immunocompromised individuals and pregnant women are unsuited for the live-attenuated orthoflavivirus vaccines^9,13^. Previously, we have demonstrated that the delivery of the BinJV/DENV2 chimeric virus vaccine using the high-density microarray patch (HD-MAP) to the skin can significantly enhance the immune responses over traditional needle-based methods^7^. Therefore, in this study, we also explored the use of the HD-MAP as an alternative vaccine delivery method^14–18^.

The HD-MAP is a solid 1 × 1 cm HD-MAP with 5000 projections/cm^2^ of 250 µm in length onto which vaccine is dry coated^19^. The HD-MAP is applied to the skin using a custom applicator at 20 m/s^14^. The dynamic delivery of the vaccine using the patch causes localised cell death by the microneedles and the delivery of the vaccines to the layers of the skin rich in antigen-presenting cells^20^. These factors contribute to enhanced immune responses and dose sparing of the vaccine administered. In several pre-clinical and clinical studies by our group, the HD-MAP has been demonstrated to be a physical immune enhancer for skin-based delivery of different vaccine types, such as inactivated, DNA, subunit and virus-like particles^14–17,19,21–30^. Apart from dose sparing, HD-MAP also provides the advantage of vaccine stability with a reduction in dependence on cold chain, possibly increasing vaccine accessibility, especially in resource-limited settings lacking appropriate cold chain infrastructure^23,31–34^.

Previously, we’ve demonstrated that the BinJV/DENV2 (bDENV2) chimeric virus-induced potent neutralising responses from a single HD-MAP dose^7^. These virus-neutralising antibody responses afforded complete protection against DENV2 challenge in the AG129 dengue mouse model^7^. Here, we expanded our studies to include all four serotypes as a monovalent and tetravalent formulation delivered by HD-MAP or intramuscular (IM) injection.

## Results

### bDENV1–4 vaccine production and quantification

**Fig. 1:**
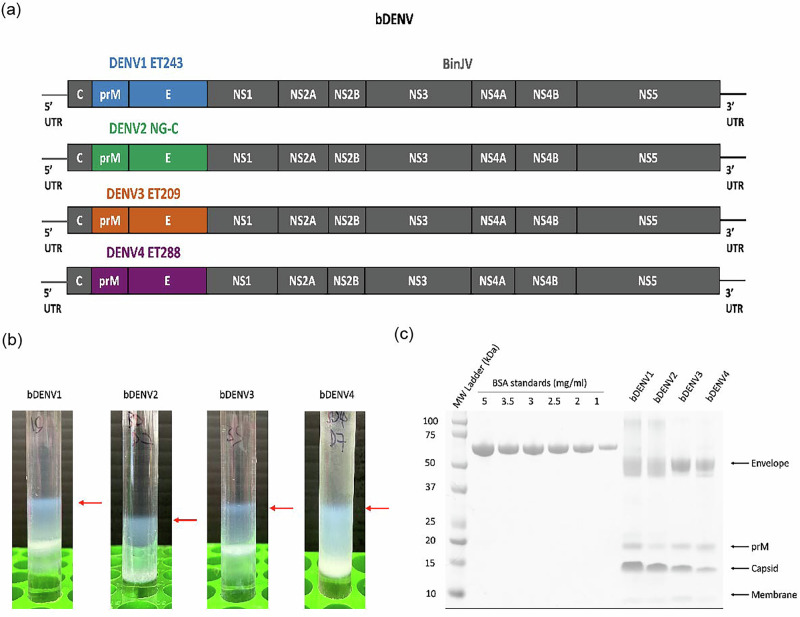
bDENV1–4 vaccine antigen design, production and analysis. **a** bDENV1–4 genome schematics with the prME genes of DENV-1 (ET243; blue), DENV-2 (NG-C; green), DENV-3 (ET209; orange) and DENV-4 (ET288; purple) were inserted into the BinJV backbone (in grey) to generate the chimeric viruses. **b** bDENV1–4 were purified via a potassium tartrate gradient, sedimenting as a wide blue opalescent band. **c** SDS-PAGE (4–12%) analysis of the gradient-purified chimeric viruses following Coomassie Blue staining. BSA standards of different concentrations (5, 3.5, 3, 2.5, 2 and 1 mg/mL) were run alongside the purified virus samples to produce a standard curve for determining the concentration of the E protein of the purified chimeric viruses. Orthoflavivirus structural proteins (prM, C, M and E protein) are as indicated. Gels were derived from the same experiment and processed in parallel.

The discovery of the new ISF, BinJV, has led to the development of chimeric viruses that can be potentially used as orthoflavivirus vaccine candidates. The chimeras were generated by inserting the prME genes of wild-type DENV-1 (ET243), DENV-2 (NG-C), DENV-3 (ET209) and DENV-4 (ET288) into the BinJV backbone (Fig. 1a). bDENV1, 2 and 4 have previously been characterised and have been found to be structurally authentic^9^. In this study, the four chimeric viruses were prepared and purified through a potassium tartrate gradient, yielding opalescent blue bands indicating purified virus in the preparation (Fig. 1b). The purity and concentration of the virus preparations were determined through SDS-PAGE analysis followed by comparative densitometry. Staining of the SDS-PAGE gel revealed bands that corresponded to the correct molecular weight for pr, M and E for all 4 dengue chimeras (Fig. 1c). These purified chimeric viruses were used for all subsequent immunisation studies.

### Vaccine coating and HD-MAP delivery

**Fig. 2:**
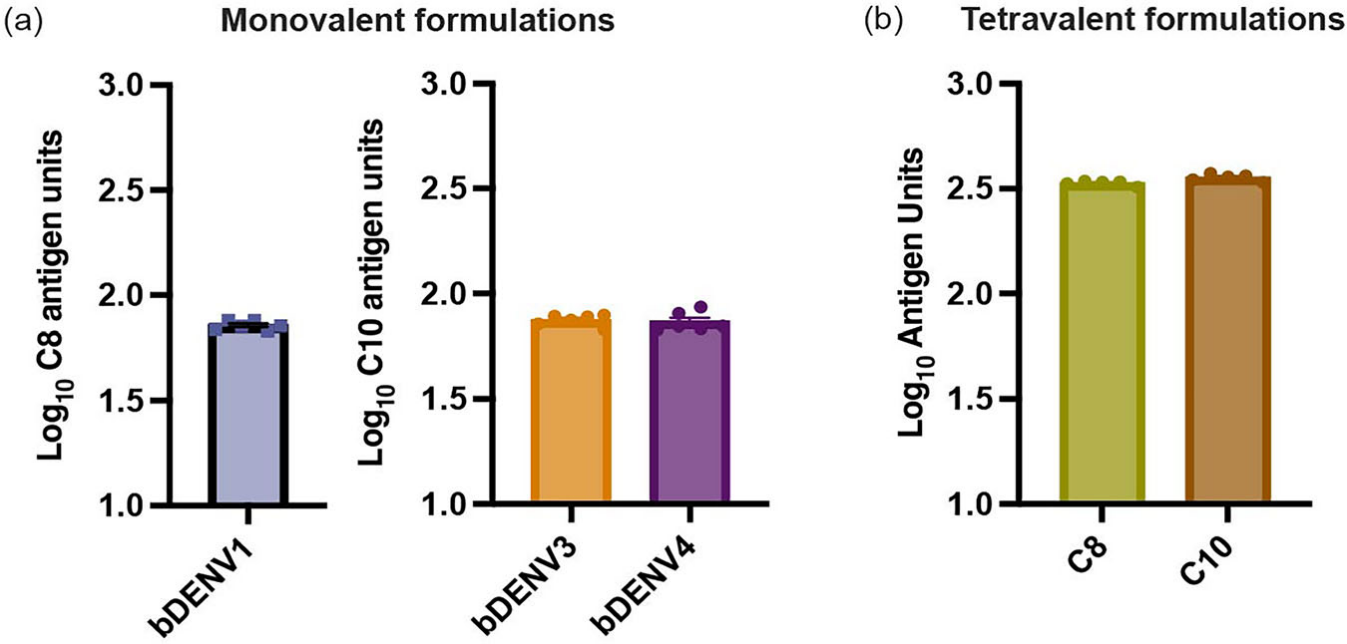
Delivery efficiencies of monovalent and tetravalent formulated bDENV1–4 coated on the HD-MAP. **a** C8 antigen capture ELISA was performed to determine the dose delivered for bDENV1 monovalent vaccine formulation, while a C10 antigen capture ELISA was performed to assess the dose delivered for bDENV3 and 4 monovalent vaccine formulations. **b** Both C8 and C10 antigen capture ELISA were performed to determine the overall amount delivered for bDENV1–4 tetravalent vaccine formulation. The bar graph represents the mean of each vaccine formulation (*n* = 5) delivered by HD-MAP, with error bars indicating the standard error of the mean.

Before performing immunisations with the HD-MAP, the delivery of the desired dose (1 µg for monovalent formulation and 4 µg for tetravalent formulation) had to be determined. We only performed delivery efficiencies for bDENV1, 3, 4 and the tetravalent formulation, as vaccine delivery conditions have been previously determined for bDENV2^7^. To quantify the amount of vaccine delivered, we measured the remaining vaccine on the microprojections of the HD-MAP post-application using a virus-capture ELISA for bDENV2. This virus-capture ELISA uses a quaternary-epitope-specific monoclonal antibody, C8, to capture the virus and 513 as the detection antibody^26^. However, due to binding efficiency differences between the different DENV serotypes, the ELISA was re-optimised for each virus using two different capture antibodies, C8 or C10 (Supplementary Fig. 1)^35,36^. C8 and C10 were used as a unit of measure and defined as the amount of vaccine displaying the quaternary epitope conformation. The C8/10 antigen was calibrated against an internal reference standard of stock bDENV1, 3 or 4 produced in-house. One C8/10 antigen unit contained 31.25 ng of bDENV1, 3 or 4. The dose of vaccine delivered was back calculated from antigen units to be 1.2, 1.3 and 1.3 μg for bDENV1, 3 and 4, respectively (Fig. 2a). As the tetravalent formulation combines all four bDENV chimeric viruses on a single HD-MAP, both C8 and C10 capture ELISA were performed to evaluate the delivery efficiencies. The dose of vaccine delivered was calculated to be 5.0 and 5.5 μg of bDENV1–4 for C8 and C10 capture ELISA, respectively (Fig. 2b).

### Immune response following monovalent vaccination

**Fig. 3:**
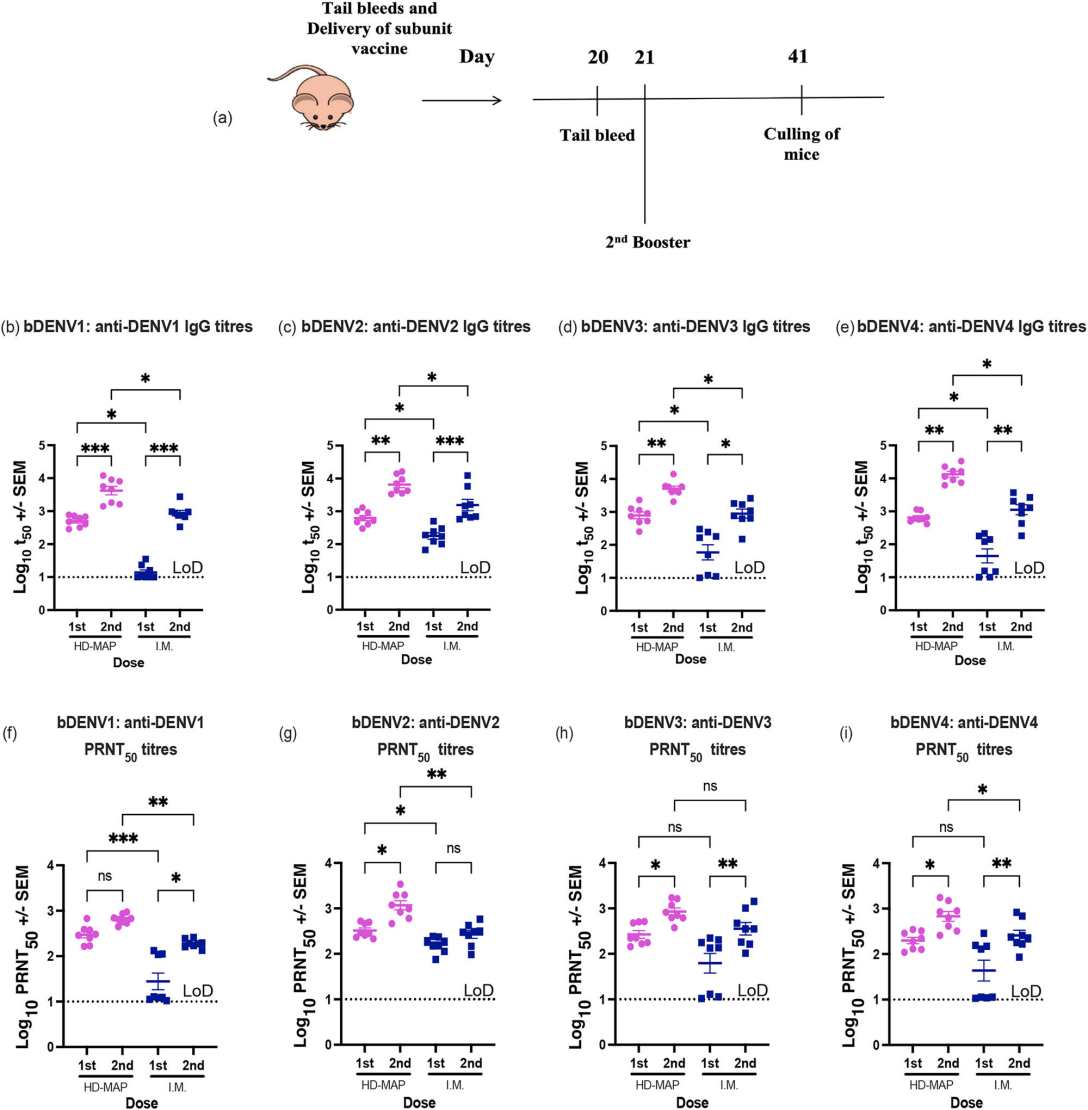
Immunisation timeline and anti-DENV IgG and neutralising responses against homologous DENV serotypes post monovalent vaccination. **a** Female BALB/c mice (*n* = 8) were vaccinated by HD-MAP or IM injection with two doses of 1 µg of bDENV1, bDENV2, bDENV3 or bDENV4, each 21 days apart. Sera obtained after the first and second immunisation were evaluated for IgG and virus-neutralising responses against homologous virus serotypes. IgG responses were plotted as mid-point antibody titres (t_50_). Scatter plot graphs represent **b** anti-DENV-1 IgG titres of bDENV1 vaccinated groups; **c** anti-DENV-2 IgG titres of bDENV2 vaccinated groups; **d** anti-DENV-3 IgG titres of bDENV3 vaccinated groups; and **e** anti-DENV-4 IgG titres of bDENV4 vaccinated groups. Neutralising antibody titres were expressed as PRNT_50_ against DENV-1 ET243, DENV-2 ET300, DENV-3 ET209 and DENV-4 ET288. Scatter plot graphs represent **f** anti-DENV-1 PRNT_50_ titres of bDENV1 vaccinated groups; **g** anti-DENV-2 PRNT_50_ titres of bDENV2 vaccinated groups; **h** anti-DENV-3 PRNT_50_ titres of bDENV3 vaccinated groups; and **i** anti-DENV-4 PRNT_50_ titres of bDENV4 vaccinated groups. Each symbol represents a single mouse. The dotted line represents the limit of detection. Lines indicate mean antibody titres with bars showing ±standard error of the mean. ****p ≤ 0.0001, ***p ≤ 0.0002, **p ≤ 0.0021, *p ≤ 0.0332, ns *p* ≤ 0.1234 assessed by Kruskal–Wallis test (*α*-level 0.05).

Following the successful demonstration of protection from HD-MAP bDENV2 in our previous study^7^, we evaluated the immunogenicity of bDENV1–4 as a monovalent vaccine delivered by HD-MAP or IM injection in a two-dose regimen. BALB/c mice were immunised with two doses of 1 μg bDENV1, 2, 3 or 4 via HD-MAP or IM and sera collected were analysed for virus-specific IgG and neutralising antibodies against all four DENV serotypes (Fig. 3a). In this study, the IM control group was administered antigen without adjuvant, allowing for a direct comparison of immune responses induced by the HD-MAP platform relative to a baseline, non-adjuvanted IM injection. This design enables the assessment of intrinsic immunostimulatory properties of the HD-MAP delivery, independent of external factors such as adjuvants, which are commonly included in clinical vaccines.

Evaluation of serotype-reactive IgG response revealed similar trends across all four serotypes. After a single dose, total IgG titres were observed to be significantly higher across all HD-MAP vaccinated group regardless of serotypes after both first (DENV-1: *p* = 0.0456; DENV-2: *p* = 0.0621; DENV-3: *p* = 0.0219; and DENV-4: *p* = 0.033) and second dose (DENV-1: *p* = 0.659; DENV-2: *p* = 0.1207; DENV-3: *p* = 0.0142; and DENV-4: *p* = 0.0289) when compared to the IM vaccinated groups (Fig. 3b–e). Similarly, these sera were also observed to have significantly higher cross-reactive IgG responses in the HD-MAP groups after a second dose (Supplementary Fig. 2).

**Fig. 4:**
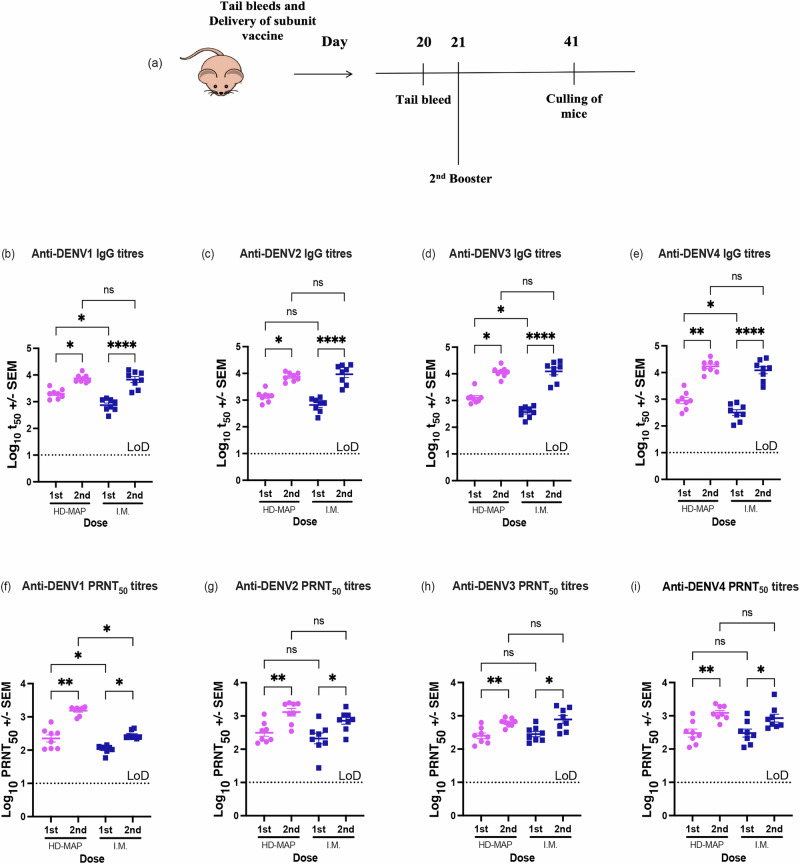
Immunisation timeline and anti-DENV IgG and neutralising responses against homologous DENV serotypes post-tetravalent vaccination. **a** Female BALB/c mice (*n* = 8) were vaccinated by HD-MAP or IM injection with two doses of 4 µg of bDENV1–4 in a tetravalent formulation, each 21 days apart. Sera obtained after the first and second immunisation were evaluated for IgG and virus-neutralising responses against homologous virus serotypes. IgG responses were plotted as mid-point antibody titres (t50). Scatter plot graphs represent **b** anti-DENV-1 IgG titres; **c** anti-DENV-2 IgG titres; **d** anti-DENV-3 IgG titres; and **e** anti-DENV-4 IgG titres. Neutralising antibody titres were expressed as PRNT_50_ against DENV-1 ET243, DENV-2 ET300, DENV-3 ET209 and DENV-4 ET288. Scatter plot graphs represent **f** anti-DENV-1 PRNT_50_ titres; **g** anti-DENV-2 PRNT_50_ titres; **h** anti-DENV-3 PRNT_50_ titres; and **i** anti-DENV-4 PRNT_50_ titres. Each symbol represents a single mouse. The dotted line represents the limit of detection. Lines indicate mean antibody titres with bars showing ±standard error of the mean. *p* ≤ 0.0001, ***p ≤ 0.0002, **p ≤ 0.0021, *p ≤ 0.0332, ns *p* ≤ 0.1234 assessed by Kruskal–Wallis test (*α*-level 0.05).

To demonstrate the functionality of the induced immune response, we assessed the serum for its neutralising abilities against all four serotypes of wild-type DENV. While virus-neutralising antibodies were induced for all HD-MAP vaccinated groups, only the bDENV2 IM group seroconverted fully following a single dose (Fig. 3g). Virus-neutralising titres were significantly higher for all HD-MAP vaccinated groups (DENV-1: *p* = 0.0034; DENV-2: *p* = 0.0051; DENV-3: *p* = 0.0428; and DENV-4: *p* = 0.00353) when compared to their IM counterparts after a single dose. Comparable cross-neutralising antibody levels were observed against heterologous serotypes (Fig. 4, Supplementary Table 1), albeit lower than their homologous serotypes (Fig. 3).

### Immune response after bDENV1–4 tetravalent vaccinations

Following the promising results from the monovalent dengue vaccination study, we next evaluated the immunogenicity of a tetravalent formulation of bDENV1–4. Female BALB/c mice were vaccinated with two doses of 4 μg of bDENV1–4 (1 μg of each bDENV serotype) by HD-MAP or IM injection (Fig. 4a), and mouse sera collected were analysed for virus-specific IgG and neutralising antibodies against all four DENV serotypes.

All animals seroconverted regardless of delivery method or DENV serotype with a single dose. Apart from DENV2, HD-MAP vaccinated groups were observed to have significantly higher IgG titres than the IM groups (DENV-1: *p* = 0.0104, Fig. 4b; DENV-3: *p* = 0.0786, Fig. 4d; and DENV-4: *p* = 0.1016, Fig. 4e). Following a second dose, IgG responses against all DENV serotypes were successfully boosted. Although the IM vaccinated group had significantly lower IgG responses after a single dose, upon boosting, the IgG titre increased, and no significant differences were observed between the two delivery routes. However, we did not observe any significant differences between virus-neutralising responses from the HD-MAP vaccinated and IM vaccinated groups, except for anti-DENV-1 responses (*p* = 0.1098, Fig. 4f) following the first vaccination. After administering the second dose, virus-neutralising antibody titres were significantly boosted against all dengue serotypes (Fig. 4).

## Discussion

Dengue is the most prevalent mosquito-borne viral disease in the world. The development of an effective dengue vaccine that reduces the burden of dengue-associated disease has been considered a high priority by the WHO for the past 50 years^37^. Here, we characterise the antibody responses to a dengue chimeric virus vaccine candidate as a monovalent and tetravalent vaccine formulation. We demonstrate that these chimeric viruses are capable of eliciting neutralising antibodies against both homologous and heterologous DENV serotypes. In addition, when coupled with the HD-MAP, enhanced antibody kinetics are observed, resulting in higher levels of neutralising antibodies elicited after a single dose.

While the licensure of Dengvaxia was a major advancement for dengue vaccine development, the limited efficacy and implementation of this vaccine resulted in a need for alternative vaccines^38–40^. To date, there are two leading vaccine candidates, TAK-003 by Takeda and TV003/005 from the National Institute of Health. TAK-003 has recently been approved for use by the European Union in individuals 4 years old and above without prior dengue infection, while they continue to pursue approvals in other dengue-endemic countries^41^. The second vaccine, TV003, has been licensed for in-country manufacturing in Brazil, Vietnam and India and also exclusively to Merck & Co in the USA^42^. The progress of these vaccine candidates marks another important development while intensive research for alternative dengue vaccines continues.

As an alternative to the live attenuated dengue vaccines, our group has been developing the chimeric bDENV platform. We have previously demonstrated that when delivered via the HD-MAP, bDENV2 produces potent immune responses that protect AG129 mice from challenge^7^. We then made the remaining three bDENV serotypes to expand upon these initial findings. As an initial proof-of-concept, these chimeric viruses are made with wild-type dengue sequences, resulting in immature virions in the prep. Our group has since then successfully designed furin-optimised bDENV viruses that can be generated for fully mature virions. For future studies, these furin-optimised bDENV viruses can be used to prevent possible ADE downstream^43^. These chimeric viruses tested as a monovalent vaccine were found to be immunogenic, raising serotype-reactive antibodies after a single immunisation with the HD-MAP. They were observed to raise strong neutralising antibody responses against homologous virus serotype following the first and second immunisation, while cross-neutralising titres against heterologous serotypes were observed to be significantly lower^44–46^. Despite observing lower levels of cross-neutralising antibodies against the heterologous serotypes, these antibodies were raised to similar levels, without having a biased response to one DENV serotype. The breadth and consistency of cross-neutralising responses observed following monovalent immunisation were particularly striking, as such balanced cross-reactivity is rarely reported even after natural primary infection. Although the underlying mechanisms remain unclear, several factors may contribute to this outcome. HD-MAP delivery induces localised cell death and strong innate immune activation, which can enhance antigen presentation and broaden the recruitment of B cell repertoires^30,47^. In addition, antigen presentation in the skin, a tissue enriched with immune cells, may favour the induction of cross-reactive responses compared with conventional IM administration.

The ideal dengue vaccine has to be tetravalent while inducing a balanced immune response against all serotypes. Vaccination with live-attenuated vaccines usually leads to immunodominance to the serotype with a higher replication rate, resulting in a need to adjust the ratio of each component^42,48,49^. With the bDENV monovalent vaccines, such immunodominance was absent as they were observed to elicit similar levels of antibodies across all four serotypes. This is most likely due to the inability of the chimeric vaccines to replicate in mammalian cells, with similar findings as the NIH’s live attenuated vaccine candidate TV003, which is also administered with the same dose for all four serotypes^50^. Hence, the tetravalent vaccine was progressed with a ratio of 1:1:1:1 of each serotype.

The tetravalent formulation on a single HD-MAP elicited a potent immune response in BALB/c mice following a single dose. This supports findings from our previous studies that the HD-MAP can elicit potent neutralising antibody responses with dose sparing compared to the traditional needle and syringe methods. The enhanced immune response observed when vaccinated using the HD-MAP is caused by a combination of the delivery of antigen and the localised cell death caused by the mechanical stress of the HD-MAP when applied to the skin^47^. Another potential explanation is that the chimeric virus particles also possess self-adjuvanting properties^51^. Preliminary evidence suggests that following vaccination, these particles may enter susceptible cells via receptor-mediated uptake and initiate the early stages of viral replication^52^. This process could generate double-stranded RNA intermediates that are sensed by innate immune receptors, leading to the induction of inflammatory cytokines that amplify the humoral response^53,54^. Although productive replication is likely impeded by factors such as intrinsic antiviral defences and suboptimal temperature conditions (37 °C), the initial innate immune activation may be sufficient to provide a strong adjuvant effect^51^. The combination of HD-MAP delivery with the chimeric vaccines elicited neutralising antibody titres from a single dose that were comparable to those achieved with two intramuscular immunisations in the monovalent study. This immune response was also observed to be balanced against all four DENV serotypes with similar neutralising antibody titres across all HD-MAP groups. However, post-second immunisation, both IgG and neutralising titres were observed to be similar across both vaccination methods. This is likely due to the selected dose of bDENV administered, resulting in a plateau of antibody responses. Beyond the scope of this study, to reveal the true dose-sparing potential of the HD-MAP, dose de-escalation studies could be performed^14,16^.

This study demonstrates the feasibility of using the BinJV/DENV chimeric platform to stably deliver multivalent flavivirus virions with strong immunogenicity. We acknowledge, however, that previous dengue vaccines based solely on prM and E proteins (e.g. Dengvaxia, purified inactivated vaccines) have shown that neutralising antibody responses alone are insufficient for long-term protection, underscoring the complexity of dengue immunity. While neutralising antibody titres were the primary readout in this study, we recognise that DENV-specific T cell responses and anti-NS1 antibodies also contribute to protective immunity. Although these were not directly measured, the BinJV/DENV platform may provide functional advantages over conventional purified or inactivated vaccines. The chimeric particles can enter susceptible cells and initiate early stages of replication, potentially exposing conserved epitopes and enhancing innate immune activation^51^. Combined with HD-MAP delivery, which induces localised cell death and robust activation of skin-resident antigen-presenting cells, this may facilitate cross-presentation and T cell priming^30^. Thus, our findings should be viewed as proof-of-concept for the platform rather than a definitive dengue vaccine strategy. We propose that this system may be most impactful for other flaviviruses, such as Zika, Japanese encephalitis, or West Nile virus, where virion-based vaccines have proven effective, or in combination approaches incorporating non-structural antigens or heterologous prime-boost regimens to broaden and sustain protective responses.

To our knowledge, this is the first paper to demonstrate effective skin delivery of a tetravalent insect-specific chimeric virus dengue vaccine. The targeted delivery of the tetravalent vaccine to the skin observed improved and balanced immunogenicity across all serotypes. The highly effective nature of the HD-MAP delivery, combined with an insect-specific chimeric virus vaccine platform, offers a promising future for the development of DENV and other orthoflaviviruses.

## Methods

### Animal ethics

Animal experiments were approved by the University of Queensland animal ethics committee (AEC No: SCMB/AIBN/322/19/NHMRC) and performed in accordance with National Health and Medical Research Council guidelines. Animals were housed in SPF conditions in the UQBR animal housing facility at the Australian Institute for Bioengineering and Nanotechnology.

### Cell lines

Vero cells (African green monkey kidney) were maintained in DMEM (Gibco) cell culture medium containing 10% foetal bovine serum (FBS, Bovogen) at 37 °C with 5% CO_2_. C6/36 (*Aedes albopictus*) cells were grown in Roswell Memorial Park Institute-1640, HEPES (RPMI, Gibco) medium, supplemented with 10% FBS, 100 U/mL penicillin (Gibco) and 100 μg/mL streptomycin (Gibco) at 28 °C.

### Generation of wild-type DENV1–4 stocks

C6/36 cells were grown to 80% confluency in T175 flasks and inoculated with wild-type DENV (DENV-1 ET243, DENV-2 ET300, DENV-3 ET209 and DENV-4 ET288) at a multiplicity of infection of 0.1 in medium containing 2% FBS. Flasks were incubated at room temperature before the virus inoculum was removed 1 h later. RPMI supplemented with 2% FBS was added, and cells were incubated at 28 °C for 3–7 days, and supernatants were harvested on days 3, 5 and 7 post-infection. Virus stocks were clarified via centrifugation at 3000 × *g* for 15 min, 4 °C and filtered through a 0.22 μM filter (Millipore) before storing at −80 °C.

### Circular polymerase extension reaction (CPER) to construct bDENV chimeric viruses

The generation of bDENV1, bDENV2 and bDENV4 chimeric viruses has previously been reported^9^. Chimeric infectious DNA constructs between BinJV (Genbank MG587038) and DENV3 (ET209, Genbank EF440434) were generated by circular polymerase extension reaction (CPER) as previously described^9^. Briefly, BinJV and DENV viral RNA were converted to cDNA using SuperScript IV reverse transcriptase (Thermo Fisher Scientific) as per the manufacturer’s instructions. The cDNAs were then used as templates for a set of primer pairs to produce six overlapping dsDNA fragments covering the BinJV backbone and the DENV3 insert. For each CPER assembly, 0.1 pmol of each viral cDNA fragment was added to a Q5 PCR (NEB). Thermal cycling conditions were as follows: [98 °C, 2 min]_x1_; [98 °C/30 s; 55 °C/30 s; 72 °C/6 min]_x2_; [98 °C/30 s; 55 °C/30 s; 72 °C/8 min]_x10_; [14 °C]_hold_. The entire CPER was transfected into C6/36 cell monolayers using Effectene transfection reagent (Qiagen) as per the manufacturer’s instructions, as the passage 0 (P_0_) cell culture supernatants were harvested 5–7 days post-transfection and stored at −80 °C.

To confirm the presence of replicating virus following CPER, preliminary analysis was performed by trypsinising P_0_ C6/36 monolayers and seeding the cells onto glass coverslips. The cells were incubated for a further 24 h prior to being fixed in 100% ice-cold acetone. Immunofluorescence assays were then performed as previously described, using the pan-orthoflavivirus reactive NS1 monoclonal antibody 4G4 (diluted 1/10)^9^.

### Propagation and purification of bDENV1–4 chimeric viruses

The propagation and purification of bDENV1–4 chimeric viruses were performed as previously described by Hobson-Peters et al.^7,9^.

### SDS-PAGE analysis of purified bDENV1–4 chimeric viruses

Purified viruses were separated on a 4–12% Bis-Tris gel to confirm the production, purity and molecular weight of viral proteins and was performed as previously described by Choo et al.^7^.

### HD-MAP coating and application

HD-MAPs (1 cm^2^, 5000 projections/cm^2^ at 250 µM in length) were provided by Vaxxas Pty. Ltd. HD-MAPs were oxygen-plasma treated for 5 min at the Australian National Fabrication Facility before vaccine coating. The coating solution for each patch was made up of 0.75% methylcellulose, 1% human serum albumin (HSA) and purified bDENV1, 2, 3 or 4 (monovalent formulation) or bDENV1–4 (tetravalent formulation) and made up to 21 µL with low-salt buffer (20 mM Tris and 75 mM NaCl, pH 7.4). 21 µL of the coating solution was added onto each patch and dry-coated on the microprojections using conditions previously described by Choo et al.^7^. Vaccine-coated HD-MAPs were then applied to the mice’s flanks using a spring-loaded applicator at a velocity of 20 m/s.

### C8 and C10 antigen capture Enzyme-Linked Immunosorbent Assay (ELISA)

The C8 and C10 antigen capture ELISA were used to analyse reconstituted samples to determine the vaccine dose delivered to the mice’s flanks post HD-MAP application. The C8 antigen capture ELISA was performed as described previously^26^ Briefly, 50 µL of a 2 µg/mL solution of humanised DENV antibody C8 or C10 in bicarbonate buffer was coated on ELISA plates (Nunc Maxisorb, Thermo Fisher) overnight at 4 °C. Coating solution was removed, and plates were blocked with blocking buffer (KPL, SeraCare) for 1 h at room temperature. Standard curves were generated by titrating 1 µg/mL of bDENV1, 2, 3 or 4 two-fold across the plate. 50 µL/well of reconstituted samples and standard curve were then added to the plate and incubated at 37 °C for 1 h. Plates were then washed with phosphate buffer saline-0.05% Tween 20 (PBS-T). Humanised DENV antibody 513 (h513)^55^ was conjugated with horse-radish peroxidase (HRP) using Abcam Lightning Link HRP conjugation kit per the manufacturer’s instructions. 50 µL of HRP-linked h513 was added to each well and incubated for an hour at 37 °C. Plates were washed with PBS-T six times, and 50 µL of Tetramethylbenzidine-w (TMBW, BioFX) was added. Relative absorbance was read at 450 nm on a Varioskan LUX multimode plate reader (Thermo Fisher).

### bDENV1–4 monovalent and tetravalent immunisation study

Female BALB/c mice (6–8 weeks old) were divided into ten groups of eight each: immunised by HD-MAP or IM injections with 1 µg of bDENV1, bDENV2, bDENV3, bDENV4 (monovalent formulation) or 4 µg of bDENV1–4 (tetravalent formulation; 1 µg of each bDENV serotype). All mice were immunised twice, each 21 days apart. Tail bleeds were obtained on day 0 and day 21, and cardiac bleeds were taken on day 42 post-first vaccination. Serum fractions were recovered by allowing the blood to clot overnight at 4 °C, followed by centrifugation at 10,000 × *g* for 10 min before storage at −20 °C until analysis.

### DENV-specific IgG ELISA

Assessment of seroconversion to the vaccine candidates was performed as described previously^7^ with the following changes. Briefly, ELISA plates were coated with 80 ng/well of bDENV1, 2, 3 or 4 in PBS overnight at 4 °C. Plates were then blocked with blocking buffer for 1 h at room temperature. Mouse sera obtained were diluted 1:100 and then serially diluted 5-fold in a round-bottom 96-well plate in blocking buffer. 50 µL/well of diluted mouse sera were added to the ELISA plate and incubated at 37 °C for 1 h. Plates were washed with PBS-T 4x and then probed with 50 µL/well of HRP-labelled goat anti-mouse antibody at a dilution of 1:1000. Plates were then washed again 6x with PBS-T before adding 50 µL/well of TMBW. Relative absorbance was read as described above.

### Plaque reduction neutralisation test (PRNT)

PRNTs were performed as described by Choo et al.^7^ with the following changes below. Briefly, mouse sera obtained on day 21 and day 42 post-first vaccination were heat-inactivated at 56 °C for 30 min and diluted 1:25, followed by a 2-fold serial dilution in a round-bottom 96-well plate in serum-free OPTI-MEM. An equal volume of DENV-1 ET243, DENV-2 ET300, DENV-3 ET209 or DENV-4 ET288 virus stock was added at 75 pfu/well to the sera dilution. The virus-serum mixture was allowed to incubate for 1 h at 37 °C before adding 50 μL to the confluent Vero cells. The remaining steps of the PRNT and immunofluorescence staining were performed as described by Choo et al.^7^ and plaques were counted to determine neutralisation titres.

### Statistical analysis

All statistical analyses were performed using GraphPad Prism version 9.0 (San Diego, CA, USA). Kruskal–Wallis test was performed for multiple comparison analysis with a 0.05 *α* level. One-way analysis of variance was performed for multiple comparison analysis with a 0.05 *α* level with a Tukey post-test.

## Supplementary information

Supplementary Figures

## Data Availability

The authors declare that all data supporting the findings of this study are available within the paper and its Supplementary Information files.
