# Supplementary material for: Tetravalent microprojection-based dengue chimeric virus vaccine raises potent neutralising antibodies in mice
Source: NPJ Vaccines. 2025 Nov 21;10:245. doi: 10.1038/s41541-025-01297-5 (PMC12638920; doi:10.1038/s41541-025-01297-5)
Supplement: Supplementary file 1 — Supplementary Figures [file 41541_2025_1297_MOESM1_ESM.pdf]

Supplementary Figures

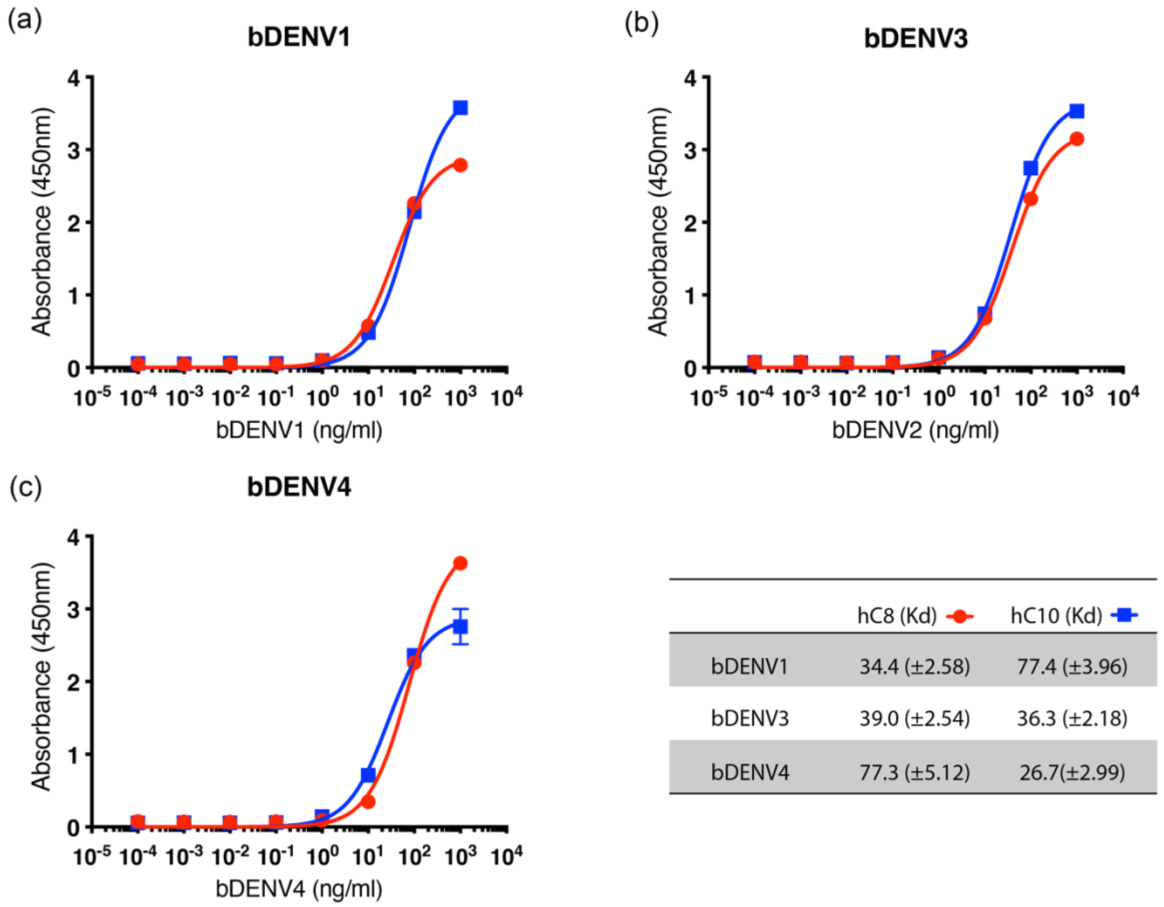

	hC8 (Kd)	hC10 (Kd)
bDENV1	34.4 (±2.58)	77.4 (±3.96)
bDENV3	39.0 (±2.54)	36.3 (±2.18)
bDENV4	77.3 (±5.12)	26.7(±2.99)

Supplementary Figure 1. Capture ELISA optimisation for bDENV1, 3 and 4. (a) ELISA curves of bDENV1, **(b)** bDENV3 and **(c)** bDENV4. Capture ELISA was performed with two different capture antibodies C8 (red) and C10 (blue) and the same detection antibody h513 to determine the antibody that best captures bDENV1, 3 or 4. Table shows legend and Kd values in nM with standard error in parenthesis, calculated using one-site specific model on Graphpad Prism 9.

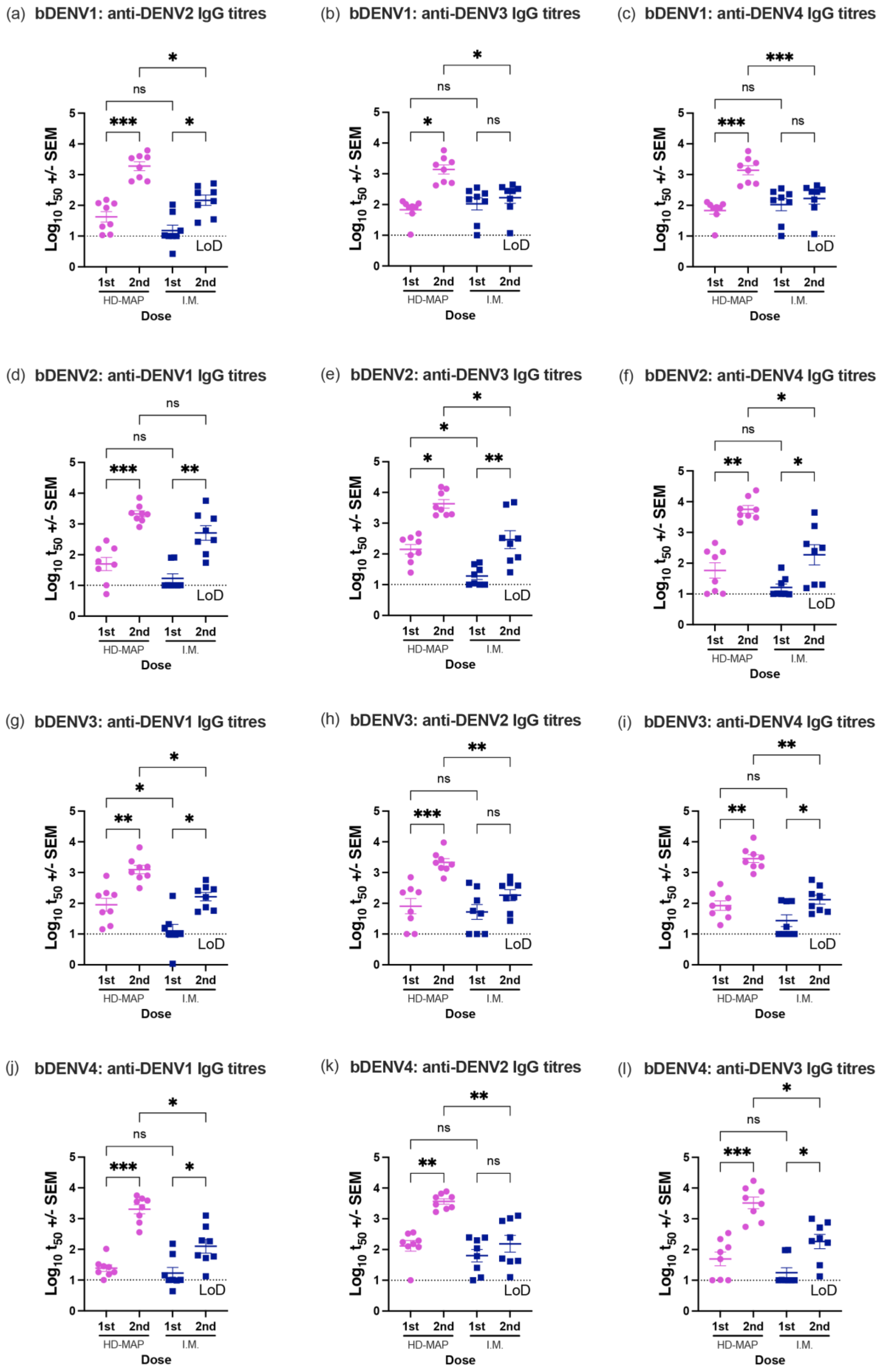
(a) bDENV1: anti-DENV2 IgG titres
(b) bDENV1: anti-DENV3 IgG titres
(c) bDENV1: anti-DENV4 IgG titres
(d) bDENV2: anti-DENV1 IgG titres
(e) bDENV2: anti-DENV3 IgG titres
(f) bDENV2: anti-DENV4 IgG titres
(g) bDENV3: anti-DENV1 IgG titres
(h) bDENV3: anti-DENV2 IgG titres
(i) bDENV3: anti-DENV4 IgG titres
(j) bDENV4: anti-DENV1 IgG titres
(k) bDENV4: anti-DENV2 IgG titres
(l) bDENV4: anti-DENV3 IgG titres
Log10 t50 +/- SEM
1st
2nd
HD-MAP
I.M.
Dose
LoD
ns

Supplementary Figure 2. Anti-DENV IgG responses against heterologous DENV serotypes post monovalent vaccination. Female BALB/c mice (*n* = 8) were vaccinated by HD-MAP or IM injection with two doses of 1 µg of bDENV1, 2, 3 or 4, each 21 days apart. Sera obtained after the first and second immunisation were evaluated for IgG responses against heterologous virus serotypes. IgG responses were plotted as mid-point antibody titres (t50). Scatter plot graphs represents **(a)** bDENV1:Anti-DENV-2 IgG titres; **(b)** bDENV1:Anti-DENV-3 IgG titres; **(c)** bDENV1:Anti-DENV-4 IgG titres; **(d)** bDENV2:Anti-DENV-1 IgG titres; **(e)** bDENV2:Anti-DENV-3 IgG titres; **(f)** bDENV2:Anti-DENV-4 IgG titres; **(g)** bDENV3:Anti-DENV-1 IgG titres; **(h)** bDENV3:Anti-DENV-2 IgG titres; **(i)** bDENV3:Anti-DENV-4 IgG titres; **(j)** bDENV4:Anti-DENV-1 IgG titres; **(k)** bDENV4:Anti-DENV-2 IgG titres; **(l)** bDENV4:Anti-DENV-3 IgG titres; Each symbol represents a single mouse. Dotted line represents limit of detection. Lines indicate mean antibody titres with bars showing +/- standard error of the mean. **** $p \leq 0.0001$, *** $p \leq 0.0002$, ** $p \leq 0.0021$, * $p \leq 0.0332$, ns $p \leq 0.1234$ assessed by Kruskal-Wallis test (α-level 0.05).

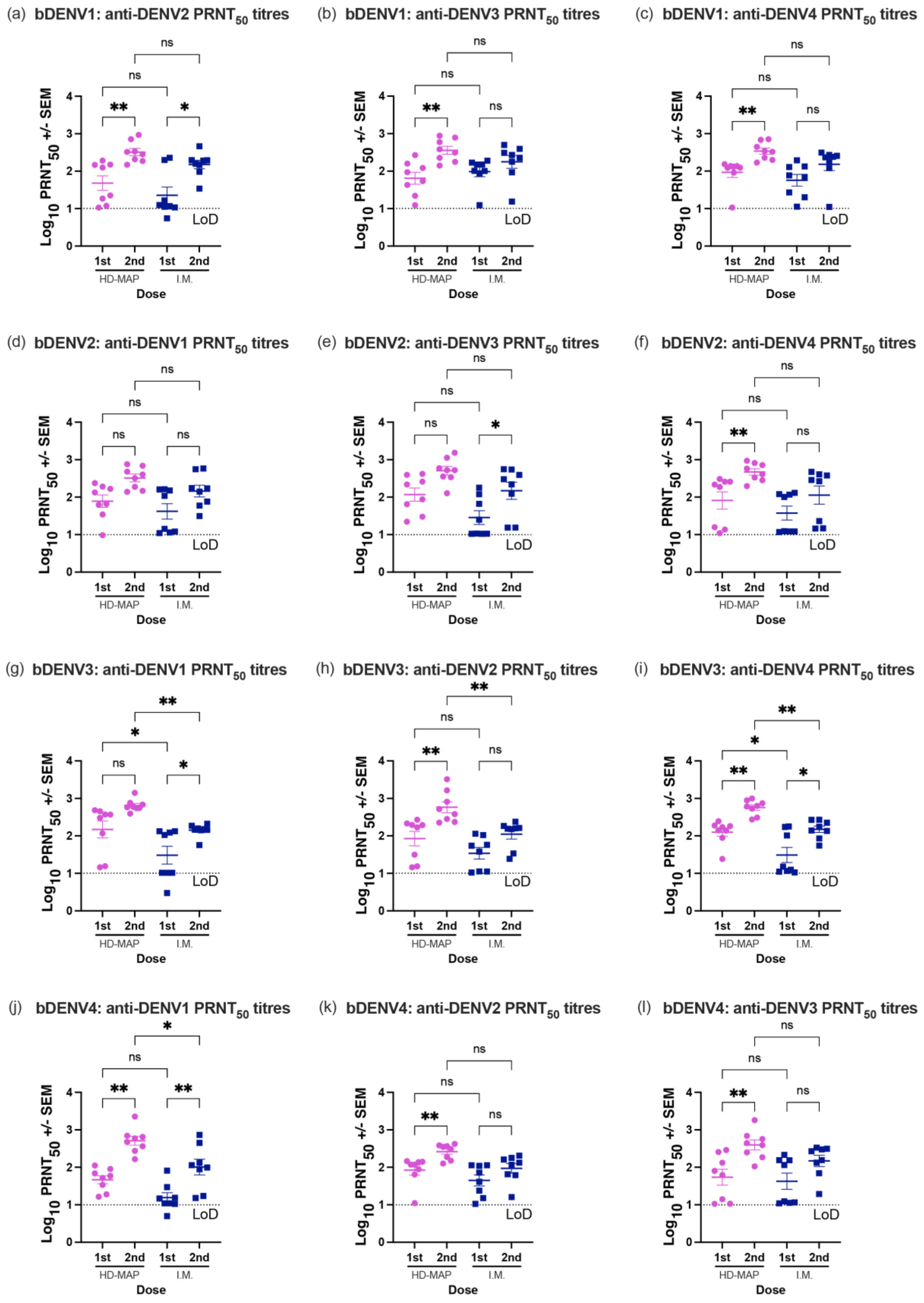

Supplementary Figure 3. Anti-DENV neutralising responses against heterologous DENV serotypes post monovalent vaccination. Female BALB/c mice (n = 8) were vaccinated by HD-MAP or IM injection with two doses of 1 µg of bDENV1, 2, 3 or 4, each 21 days apart. Sera obtained after the first and second

immunisation were evaluated for virus-neutralising responses against heterologous virus serotypes. Neutralising antibody titres were expressed as $PRNT_{50}$ against DENV-1 ET243, DENV-3 ET209 and DENV-4 ET288. Scatter plot graphs represents **(a)** bDENV1:Anti-DENV-2 $PRNT_{50}$ titres; **(b)** bDENV1:Anti-DENV-3 $PRNT_{50}$ titres; **(c)** bDENV1:Anti-DENV-4 $PRNT_{50}$ titres; **(d)** bDENV2:Anti-DENV-1 $PRNT_{50}$ titres; **(e)** bDENV2:Anti-DENV-3 $PRNT_{50}$ titres; **(f)** bDENV2:Anti-DENV-4 $PRNT_{50}$ titres; **(g)** bDENV3:Anti-DENV-1 $PRNT_{50}$ titres; **(h)** bDENV3:Anti-DENV-2 $PRNT_{50}$ titres; **(i)** bDENV3:Anti-DENV-4 $PRNT_{50}$ titres; **(j)** bDENV4:Anti-DENV-1 $PRNT_{50}$ titres; **(k)** bDENV4:Anti-DENV-2 $PRNT_{50}$ titres; **(l)** bDENV4:Anti-DENV-3 $PRNT_{50}$ titres; Each symbol represents a single mouse. Dotted line represents limit of detection. Lines indicate mean antibody titres with bars showing +/- standard error of the mean. **** $p \leq 0.0001$, *** $p \leq 0.0002$, ** $p \leq 0.0021$, * $p \leq 0.0332$, ns $p \leq 0.1234$ assessed by Kruskal-Wallis test (α-level 0.05).

Supplementary Table 1. ANOVA analysis of heterologous virus neutralising titres post 2nd dose of monovalent vaccination

**BinJV/DENV1**				
	**Mean Diff.**	**95.00% CI of diff.**	**Summary**	**Adjusted P Value**
**anti-DENV1 vs. anti-DENV2**	**0.3058**	**-0.3841 to 0.9956**	**ns**	**0.9731**
**anti-DENV1 vs. anti-DENV3**	**0.2596**	**-0.4303 to 0.9495**	**ns**	**0.9944**
**anti-DENV1 vs. anti-DENV4**	**0.2816**	**-0.4083 to 0.9715**	**ns**	**0.9874**
**anti-DENV1 vs. anti-DENV2 (IM)**	**0.09825**	**-0.5916 to 0.7881**	**ns**	**>0.9999**
**anti-DENV1 vs. anti-DENV3 (IM)**	**0.02912**	**-0.6608 to 0.7190**	**ns**	**>0.9999**
**anti-DENV1 vs. anti-DENV4 (IM)**	**0.093**	**-0.5969 to 0.7829**	**ns**	**>0.9999**
**BinJV/DENV2**				
	**Mean Diff.**	**95.00% CI of diff.**	**Summary**	**Adjusted P Value**
**anti-DENV2 vs. anti-DENV1**	**-0.5579**	**-1.355 to 0.2396**	**ns**	**0.5136**
**anti-DENV2 vs. anti-DENV3**	**0.3651**	**-0.4323 to 1.163**	**ns**	**0.9642**
**anti-DENV2 vs. anti-DENV4**	**0.3969**	**-0.4006 to 1.194**	**ns**	**0.9298**
**anti-DENV2 vs. anti-DENV1 (IM)**	**-0.2686**	**-1.066 to 0.5288**	**ns**	**0.9983**
**anti-DENV2 vs. anti-DENV3 (IM)**	**0.2591**	**-0.5383 to 1.057**	**ns**	**0.9989**

**anti-DENV2 vs. anti-DENV4 (IM)**	**0.3755**	**-0.4219 to 1.173**	**ns**	**0.9548**
**BinJV/DENV3**				
	**Mean Diff.**	**95.00% CI of diff.**	**Summary**	**Adjusted P Value**
**anti-DENV3 vs. anti-DENV1**	**-0.1206**	**-0.8644 to 0.6232**	**ns**	**>0.9999**
**anti-DENV3 vs. anti-DENV2**	**-0.1671**	**-0.9109 to 0.5767**	**ns**	**>0.9999**
**anti-DENV3 vs. anti-DENV4**	**0.1738**	**-0.5701 to 0.9176**	**ns**	**>0.9999**
**anti-DENV3 vs. anti-DENV1 (IM)**	**-0.4023**	**-1.146 to 0.3416**	**ns**	**0.8723**
**anti-DENV3 vs. anti-DENV2 (IM)**	**-0.5094**	**-1.253 to 0.2344**	**ns**	**0.5511**
**anti-DENV3 vs. anti-DENV4 (IM)**	**0.3772**	**-0.3666 to 1.121**	**ns**	**0.9191**
**BinJV/DENV4**				
	**Mean Diff.**	**95.00% CI of diff.**	**Summary**	**Adjusted P Value**
**anti-DENV4 vs. anti-DENV1**	**-0.1208**	**-0.8644 to 0.6229**	**ns**	**>0.9999**
**anti-DENV4 vs. anti-DENV2**	**-0.4104**	**-1.154 to 0.3333**	**ns**	**0.8541**
**anti-DENV4 vs. anti-DENV3**	**-0.2316**	**-0.9753 to 0.5120**	**ns**	**0.9993**
**anti-DENV4 vs. anti-DENV1 (IM)**	**-0.4044**	**-1.148 to 0.3393**	**ns**	**0.8676**
**anti-DENV4 vs. anti-DENV2 (IM)**	**-0.439**	**-1.183 to 0.3046**	**ns**	**0.7799**

**anti-DENV4 vs. anti-DENV3 (IM)**	**-0.24**	**-0.9836 to 0.5037**	**ns**	**0.9989**
